# Identification of metastatic primary cutaneous squamous cell carcinoma utilizing artificial intelligence analysis of whole slide images

**DOI:** 10.1038/s41598-022-13696-y

**Published:** 2022-06-14

**Authors:** Jaakko S. Knuutila, Pilvi Riihilä, Antti Karlsson, Mikko Tukiainen, Lauri Talve, Liisa Nissinen, Veli-Matti Kähäri

**Affiliations:** 1 Department of Dermatology, University of Turku and Turku University Hospital, Hämeentie 11 TE6, 20520 Turku, Finland; 2 FICAN West Cancer Research Laboratory, University of Turku and Turku University Hospital, Turku, Finland; 3 Auria Biobank, University of Turku and Turku University Hospital, Turku, Finland; 4 Department of Pathology, University of Turku and Turku University Hospital, Turku, Finland

**Keywords:** Squamous cell carcinoma, Prognostic markers, Risk factors

## Abstract

Cutaneous squamous cell carcinoma (cSCC) harbors metastatic potential and causes mortality. However, clinical assessment of metastasis risk is challenging. We approached this challenge by harnessing artificial intelligence (AI) algorithm to identify metastatic primary cSCCs. Residual neural network-architectures were trained with cross-validation to identify metastatic tumors on clinician annotated, hematoxylin and eosin-stained whole slide images representing primary non-metastatic and metastatic cSCCs (n = 104). Metastatic primary tumors were divided into two subgroups, which metastasize rapidly (≤ 180 days) (n = 22) or slowly (> 180 days) (n = 23) after primary tumor detection. Final model was able to predict whether primary tumor was non-metastatic or rapidly metastatic with slide-level area under the receiver operating characteristic curve (AUROC) of 0.747. Furthermore, risk factor (RF) model including prediction by AI, Clark’s level and tumor diameter provided higher AUROC (0.917) than other RF models and predicted high 5-year disease specific survival (DSS) for patients with cSCC with 0 or 1 RFs (100% and 95.7%) and poor DSS for patients with cSCCs with 2 or 3 RFs (41.7% and 40.0%). These results indicate, that AI recognizes unknown morphological features associated with metastasis and may provide added value to clinical assessment of metastasis risk and prognosis of primary cSCC.

## Introduction

Cutaneous squamous cell carcinoma (cSCC) is the most common metastatic skin cancer with increasing incidence worldwide^1,2^. The overall rate of metastasis of cSCC has been estimated as 1–4% and it accounts for at least 20% of all skin cancer-related mortality^2–4^. The prognosis of patients with metastatic disease is generally poor with 3-year overall survival (OS) of 29–46% and mortality is associated primarily with nodal metastases^3,5^. Metastasis occurs relatively rapidly with every other patient developing detectable metastasis within 6 months of the diagnosis of primary metastatic cSCC (mcSCC)^3^.

Established tumor staging systems by American Joint Committee on Cancer (AJCC) and Brigham and Women’s Hospital (BWH) are utilized in the clinical risk assessment of cSCC patients. Tumor diameter, invasion depth, and perineural invasion are associated with the risk of metastasis and are pivotal factors in both the BWH and the 8^th^ edition of AJCC (AJCC-8) staging systems^6^. However, current staging systems are unsatisfactory in predicting the risk of progression of primary cSCC to metastatic disease^7^. In addition, to date, there are no clinically established biomarkers for assessment of metastasis risk of primary cSCC.

Current digitalization of pathology, especially whole slide imaging enables utilization of machine learning (ML) in the analysis of tissue specimens^8^. ML represents a subfield of artificial intelligence (AI) and can be utilized via supervised, unsupervised, or reinforcement learning^9,10^. Supervised learning dominates medical applications and is based on training with data with known outcomes with the aim of finding a mathematical function that can map input data into output predictions^11^. In general, ML algorithms include regression and more advanced artificial neural networks (ANNs), which are computing programs inspired by biological neural networks^12^. ML is called deep learning (DL), when ANN includes multiple (> 1) hidden layers between the input and output layers and it represents more recent evolution of ML with the ability to handle large datasets^13^. DL requires less manual preprocessing and can learn more complex features with higher efficiency enabling more successful pattern recognition and computer vision^14^. Numerous specialized deep neural networks, such as convolutional neural networks (CNNs) have been developed for different tasks^15^.

CNNs are deep neural networks, which contain hidden convolutional layer(s) in the architecture^9^. Connectivity pattern between the neurons of the CNN resembles the organization of the animal visual cortex and demonstrates higher performance regarding spatial features crucial for the performance of computer vision such as object recognition, identification and classification^10^. Residual neural network-18 (ResNet-18) is an 18-layer deep and ResNet-50 a 50-layer deep CNN, which adds residual learning to the traditional CNN^16^. ResNets resemble pyramidal cells in cerebral cortex and utilize skip connections to jump over layers in order to solve the problem of gradient dispersion and accuracy degradation^16^.

To date, CNNs have been studied as diagnostic tools in clinical dermatology in dermoscopy and dermatopathology^17^. In dermatopathology, CNNs have been shown to successfully identify basal cell carcinomas, dermal nevi and seborrheic keratoses, to distinguish Spitz nevi from conventional melanocytic lesions and to predict the prognosis of primary melanoma^18–20^.

In this study, we have used AI algorithm to identify primary cSCCs with risk for metastasis. ResNet architectures were trained with cross-validation and fine-tuned on tumor tiles extracted from clinician annotated, hematoxylin and eosin-stained whole slide images representing a cohort of primary mcSCCs and a cohort of non-metastatic cSCCs (non-mcSCCs)^3^. Furthermore, a risk factor model (RFM), which utilized prediction by AI and conventional histopathological features was generated.

## Results

### Performance of AI-models

For testing of AI-models we utilized input data from 45 whole slide images (WSIs) representing mcSCCs and 59 WSIs representing non-mcSCCs. For rapid metastasis -AI-model, WSIs of a subcohort of 22 rapidly (≤ 180 days) metastatic mcSCCs was used. Tumor characteristics of the rapid metastasis -AI-model are shown in Table 1.

**Table 1:** Clinicopathological primary tumor characteristics of the final cohorts utilized in the rapid metastasis -AI-model (tumor n = 81). AI: artificial intelligence; AJCC-8: the 8th edition of American Joint Committee on Cancer; BWH: Brigham and Women’s Hospital; IQR: interquartile range; mcSCC: primary metastatic squamous cell carcinoma; WSI: whole slide image.

Baseline primary tumor characteristics	Total	Non-mcSCC	Rapid mcSCC	*P* value
**Number of samples**	81	59	22	
**Age at the day of sample**				0.115
Median, y (IQR)	78 (71–85)	79 (71–87)	76 (71–81)	
Mean, y	76	77	73	
Range, y	46–93	55–93	46–93	
**Gender**				0.216
Male, n (%)	54 (66.7)	37 (62.7)	17 (77.3)	
Female, n (%)	27 (33.3)	22 (37.3)	5 (22.7)	
**Nature of the tissue specimen**				0.479
Biopsy, n (%)	11 (13.6)	7 (11.9)	4 (18.2)	
Resection, n (%)	70 (86.4)	52 (88.1)	18 (81.8)	
**Number of local recurrences**				0.756
Primary, n (%)	74 (91.4)	54 (91.5)	20 (90.9)	
First recurrence, n (%)	6 (7.4)	4 (6.8)	2 (9.1)	
Fifth recurrence, n (%)	1 (1.2)	1 (1.7)	0 (0.0)	
**Location**				0.014
Auricle/pre-/retroauricular, n (%)	17 (21.0)	12 (20.3)	5 (22.7)	
Temple, n (%)	12 (14.8)	9 (15.3)	3 (13.6)	
Nose, n (%)	5 (6.2)	5 (8.5)	0 (0.0)	
Scalp, n (%)	9 (11.1)	8 (13.6)	1 (4.5)	
Forehead, n (%)	3 (3.7)	1 (1.7)	2 (9.1)	
Orbita, n (%)	1 (1.2)	0 (0.0)	1 (4.5)	
Lip, n (%)	5 (6.2)	1 (1.7)	4 (18.2)	
Cheek, n (%)	11 (13.6)	11 (18.6)	0 (0.0)	
Neck, n (%)	2 (2.5)	1 (1.7)	1 (4.5)	
Torso, n (%)	1 (1.2)	1 (1.7)	0 (0.0)	
Upper limp, n (%)	8 (9.9)	6 (10.2)	2 (9.1)	
Lower limb, n (%)	6 (7.4)	3 (5.1)	3 (13.6)	
Unknown, n (%)	1 (1.2)	1 (1.7)	0 (0.0)	
**Histological grade**				0.247
1, n (%)	40 (49.4)	32 (54.2)	8 (36.4)	
2, n (%)	28 (34.6)	18 (30.5)	10 (45.5)	
3, n (%)	10 (12.3)	6 (10.2)	4 (18.2)	
Unknown, n (%)	3(3.7)	3(5.1)	0(0.0)	
**Diameter**				< 0.001
< 10 mm, n (%)	24 (29.6)	22 (37.3)	2 (9.1)	
10–19.9 mm, n (%)	27 (33.3)	24 (40.7)	3 (13.6)	
20–29.9 mm, n (%)	12 (14.8)	7 (11.9)	5 (22.7)	
≥ 30 mm, n (%)	18 (22.2)	6 (10.2)	12 (54.5)	
Unknown, n (%)	0 (0.0)	0 (0.0)	0 (0.0)	
**Clark’s level**				< 0.001
2–4 tumor, n (%)	48 (59.3)	44 (74.6)	4 (18.2)	
5 tumor, n (%)	27 (33.3)	12 (20.3)	15 (68.2)	
Unknown, n (%)	6 (7.4)	3 (5.1)	3 (13.6)	
**Perineural invasion**				1.000
Not present, n (%)	80 (98.8)	58 (98.3)	22 (100.0)	
Present, n (%)	1 (1.2)	1 (1.7)	0 (0.0)	
**Lymphovascular invasion**				0.178
Not present, n (%)	78 (96.3)	58 (98.3)	20 (90.9)	
Present, n (%)	3 (3.7)	1 (1.7)	2 (9.1)	
**Invasion beyond subcutaneous fat**				< 0.001
No, n (%)	57 (70.4)	49 (83.1)	8 (36.4)	
Yes, n (%)	23 (28.4)	10 (16.9)	13 (59.1)	
Unknown, n (%)	1 (1.2)	0 (0.0)	1 (4.5)	
**Tumor staging**				
AJCC-8				< 0.001
T1, n (%)	46 (56.8)	44 (74.6)	2 (9.1)	
T2, n (%)	6 (7.4)	3 (5.1)	3 (13.6)	
T3, n (%)	24 (29.6)	9 (15.3)	15 (68.2)	
T4a–T4b, n (%)	4 (4.9)	3 (5.1)	1 (4.5)	
Unknown, n (%)	1 (1.2)	0 (0.0)	1 (4.5)	
BWH				< 0.001
T1, n (%)	37 (45.7)	36 (61.0)	1 (4.5)	
T2a, n (%)	17 (21.0)	11 (18.6)	6 (27.3)	
T2b, n (%)	20 (24.7)	7 (11.9)	13 (59.1)	
T3, n (%)	4 (4.9)	3 (5.1)	1 (4.5)	
Unknown, n (%)	3 (3.7)	2 (3.4)	1 (4.5)	
**AI prediction**				0.001
Non-metastatic, n (%)	53 (65.4)	45 (76.3)	8 (36.4)	
Metastatic, n (%)	28 (34.6)	14 (23.7)	14 (63.6)	
**Pathologist prediction**				0.003
Non-metastatic, n (%)	47 (58.0)	40 (67.8)	7 (31.8)	
Metastatic, n (%)	20 (24.7)	10 (16.9)	10 (45.5)	
Cannot be assessed, n (%)	14 (17.3)	9 (15.3)	5 (22.7)	

In single tile -AI-model, a slide level area under the receiver operating characteristic curve (AUROC) of 0.689 was reached at best. However, the model was dramatically overfitting to the training data despite heavy regulation and data augmentation and ultimately the model could not reliably reproduce the results between different sampling of folds. Invasive front -AI-model produced results inferior to the single tile -AI-model with tile level AUROC of 0.629 at best, regardless of whether tiles inside the annotation, outside the annotation or both were included. Multi-tile -AI-model did not produce more convincing results than the previous approaches (AUROC 0.672 at best) on stack tile level. This may be due to the fact that using n tiles as input effectively reduced the size of the dataset by n-fold, since we retained from showing a tile in different stacks multiple times.

**Figure 1:**
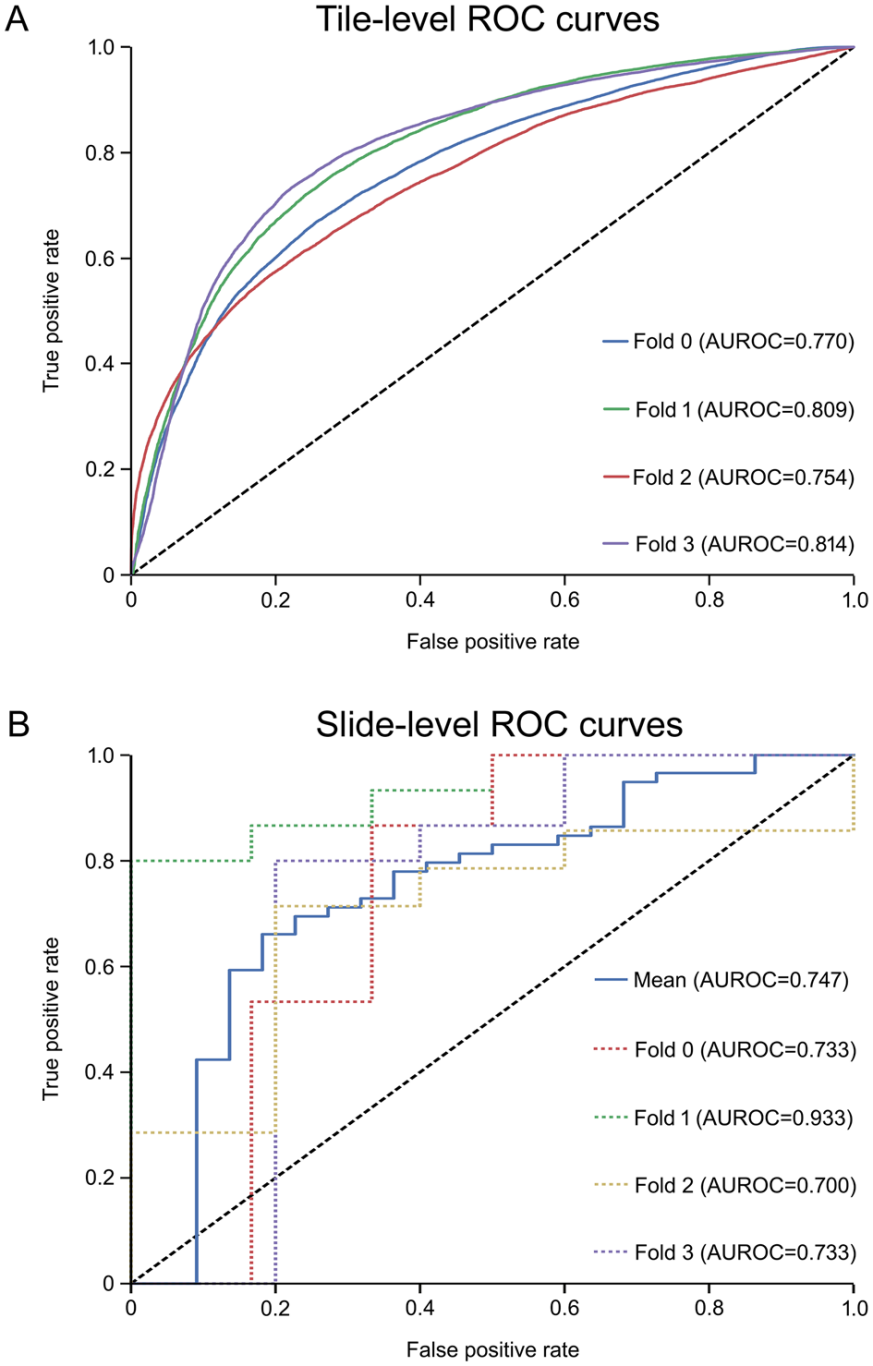
Receiver operating characteristic (ROC) curves and area under the receiver operating characteristic (AUROC) curve scores of the final rapid metastasis -AI-model. (**A**) Tile-level and (**B**) Slide-level results with different fourfold cross-validation folds are shown.

Rapid metastasis -AI-model generated most convincing results from the beginning. Finally, AUROCs of 0.754–0.814 were reached on tile level depending on the fold (Fig. 1A). Furthermore, an average AUROC of 0.747 was achieved on slide level (Fig. 1B). Slide level results visualizing summary confusion matrices show that sensitivity of the rapid metastasis -AI-model was 64%, specificity 76% and accuracy 73% (Supplementary Fig. 1). This rapid metastasis -AI-model was utilized in the creation of RFMs and survival analyses.

### Risk factor analysis and evaluation by dermatopathologist

Next, we evaluated whether traditional histopathological features could predict the risk of metastasis better than the rapid metastasis -AI-model and whether the predictive power of the model could be explained by histopathological features. Pearson correlation was conducted taking into account every variable in Table 1. The highest correlation of AI prediction (0.329) was with AJCC-8 tumor staging and second highest (0.256) with BWH tumor staging (Supplementary Table 1). In comparison, Pearson correlations regarding prediction by pathologist were higher with highest correlation of 0.494 with BWH staging system and second highest (0.415) with Clark’s level (Supplementary Table 1). Thus, it can be concluded that AI prediction did not strongly rely on any of the classical clinicopathological variables but was based on other morphological features of the tumor.

Logistic regression analysis was conducted and AUROCs created to evaluate the metastasis risk for each variable and to estimate the classification power of the rapid-metastasis -AI-model. Rapid metastasis -AI-model provided a slide level AUROC of 0.747 and an odds ratio (OR) of 5.63. In comparison, pathologist reached an AUROC of 0.694 and an OR of 5.71. Clark’s level provided higher OR (13.75) and AUROC (0.788) than invasion beyond fat, and diameter provided highest AUROC of 0.804 of the individual clinicopathological variables. AJCC-8 and BWH tumor staging systems provided slightly higher AUROCs (0.816 and 0.818, respectively), but in logistic regression analysis the increase of risk was non-linear (Table 2).

**Table 2:** Analysis of metastasis risk utilizing final rapid metastasis -AI-model cohorts. *Alternative grouping in which 20–29.9 mm and ≥ 30 mm categories are combined. AI: artificial intelligence; AI (met): analyzed by AI as metastatic; AJCC-8: The eight edition of American joint committee on cancer tumor staging; AUROC: area under receiver operating characteristic curve; BWH: Brigham and Women’s Hospital tumor staging; CI: confidence interval; Clark (5): Clark’s level 5; Diameter (≥ 30): tumor diameter ≥ 30 mm; mcSCC: primary metastatic squamous cell carcinoma; OR: odds ratio; Pathologist (met): analyzed by pathologist as metastatic; positive/total: tumors with named category out of all tumors with known information about named feature; RFM: risk factor model; ref: reference category.

Analysis of metastasis risk	Included in analyses		Risk of metastasis by variable Unadjusted OR (95% CI)	*p* value	AUROC	*p* value
	Rapid mcSCC	Non-mcSCC				
**Gender**					0.573	0.316
Male, n (positive/total) (%)	17/22 (77.3)	37/59 (62.7)	2.02 (0.65–6.25)	0.221		
Female, n (positive/total) (%)	5/22 (22.7)	22/59 (37.3)	1 (ref.)			
**Histological grade**					0.609	0.137
1, n (positive/total) (%)	8/22 (36.4)	32/56 (57.1)	1 (ref.)			
2, n (positive/total) (%)	10/22 (45.5)	18/56 (32.1)	2.22 (0.74–6.64)	0.153		
3, n (positive/total) (%)	4/22 (18.2)	6/56 (10.7)	2.67 (0.61–11.76)	0.195		
**Diameter**					0.804	< 0.001
< 10 mm, n (positive/total) (%)	2/22 (9.1)	22/59 (37.3)	1 (ref.)			
10–19.9 mm, n (positive/total) (%)	3/22 (13.6)	24/59 (40.7)	1.38 (0.21–9.02)	0.740		
20–29.9 mm, n (positive/total) (%)	5/22 (22.7)	7/59 (11.9)	7.86 (1.24–49.83)	0.029		
≥ 30 mm, n (positive/total) (%)	12/22 (54.5)	6/59 (10.2)	22.00 (3.83–126.36)	0.001		
≥ 20 mm, n (positive/total) (%)*	17/22 (77.3)	13/59 (22.0)	14.39 (2.85–72.52)	0.001		
**Clark’s level**					0.788	< 0.001
2–4, n (positive/total) (%)	4/19 (21.1)	44/56 (78.6)	1 (ref.)			
5, n (positive/total) (%)	15/19 (78.9)	12/56 (21.4)	13.75 (3.85–49.17)	< 0.001		
**Invasion beyond fat**					0.725	0.002
No, n (positive/total) (%)	8/21 (38.1)	49/59 (83.1)	1 (ref.)			
Yes, n (positive/total) (%)	13/21 (61.9)	10/59 (16.9)	7.96 (2.62–24.23)	< 0.001		
**Tumor staging**						
AJCC-8					0.816	< 0.001
T1, n (positive/total) (%)	2/21 (9.5)	44/59 (74.6)	1 (ref.)			
T2, n (positive/total) (%)	3/21 (14.3)	3/59 (5.1)	22.00 (2.60–186.53)	0.005		
T3, n (positive/total) (%)	15/21 (71.4)	9/59 (15.3)	36.67 (7.11–189.10)	< 0.001		
T4a–T4b, n (positive/total) (%)	1/21 (4.8)	3/59 (5.1)	7.33 (0.51–105.92)	0.144		
BWH					0.818	< 0.001
T1, n (positive/total) (%)	1/21 (4.8)	36/57 (63.2)	1 (ref.)			
T2a, n (positive/total) (%)	6/21 (28.6)	11/57 (19.3)	19.64 (2.13–181.18)	0.009		
T2b, n (positive/total) (%)	13/21 (61.9)	7/57 (12.3)	66.86 (7.49–596.88)	< 0.001		
T3, n (positive/total) (%)	1/21 (4.8)	3/57 (5.3)	12.00 (0.59–243.85)	0.106		
**Prediction by pathologist**					0.694	0.017
Non-metastatic, n (positive/total) (%)	7/17 (41.2)	40/50 (80.0)	1 (ref.)			
Metastatic, n (positive/total) (%)	10/17 (58.8)	10/50 (20.0)	5.71 (1.74–18.76)	0.004		
**Prediction by AI**					0.747	< 0.001
Non-metastatic, n (positive/total) (%)	8/22 (36.4)	45/59 (76.3)	1 (ref.)			
Metastatic, n (positive/total) (%)	14/22 (63.6)	14/59 (23.7)	5.63 (1.96–16.17)	0.001		
**Pathologist (met) + Clark (5)**					0.807	< 0.001
Zero risk factors, n (positive/total) (%)	2/17 (11.8)	32/48 (66.7)	1 (ref.)			
One risk factor, n (positive/total) (%)	7/17 (41.2)	11/48 (22.9)	10.18 (1.83–56.54)	0.008		
Two risk factors, n (positive/total) (%)	8/17 (47.1)	5/48 (10.4)	25.60 (4.17–157.00)	< 0.001		
**AI (met) + Clark (5)**					0.872	< 0.001
Zero risk factors, n (positive/total) (%)	0/19 (0.0)	33/56 (58.9)	NA	NA		
One risk factor, n (positive/total) (%)	11/19 (57.9)	22/56 (39.3)	NA	NA		
Two risk factors, n (positive/total) (%)	8/19 (42.1)	1/56 (1.8)	NA	NA		
**Conventional-RFM**					0.862	< 0.001
Zero risk factors, n (positive/total) (%)	2/19 (10.5)	43/56 (76.8)	1 (ref.)			
One risk factor, n (positive/total) (%)	8/19 (42.1)	10/56 (17.9)	17.20 (3.16–93.72)	0.001		
Two risk factors, n (positive/total) (%)	9/19 (47.4)	3/56 (5.4)	64.50 (9.38–443.51)	< 0.001		
**Pathologist-RFM**					0.841	< 0.001
Zero risk factors, n (positive/total) (%)	2/16 (12.5)	31/48 (64.6)	NA	NA		
One risk factor, n (positive/total) (%)	3/16 (18.8)	10/48 (20.8)	NA	NA		
Two risk factors, n (positive/total) (%)	6/16 (37.5)	7/48 (14.6)	NA	NA		
Three risk factors, n (positive/total) (%)	5/16 (31.3)	0/48 (0.0)	NA	NA		
**AI-RFM**					0.917	< 0.001
Zero risk factors, n (positive/total) (%)	0/19 (0.0)	32/56 (57.1)	NA	NA		
One risk factor, n (positive/total) (%)	5/19 (26.3)	20/56 (35.7)	NA	NA		
Two risk factors, n (positive/total) (%)	9/19 (47.4)	4/56 (7.1)	NA	NA		
Three risk factors, n (positive/total) (%)	5/19 (26.3)	0/56 (0.0)	NA	NA		

Next, it was evaluated, whether prediction as metastatic by AI could act as an individual risk factor in multifactorial risk factor model (RFM) and improve the clinical risk assessment. For comparison, a RFM (conventional-RFM) taking into account tumor diameter ≥ 30 mm and Clark’s level 5 as risk factors provided higher AUROC (0.862) than AJCC-8 or BWH staging systems. Furthermore, a RFM containing prediction by AI as metastatic and Clark’s level 5 as risk factors provided even higher AUROC of 0.872. Finally, a RFM including prediction by AI as metastatic, Clark’s level 5 and diameter ≥ 30 mm as risk factors (AI-RFM) produced an AUROC of 0.917. Similar RFMs that took into account prediction by pathologist instead of AI resulted in lower AUROCs of 0.807 and 0.841 respectively (Table 2).

Different thresholds for AI-RFM were tested with respect to diameter of primary tumor. Selecting 20 mm instead of 30 mm as a threshold for the risk factor, resulted in lower AUROC of 0.913. Furthermore, if histological grade was included in AI-RFM model with 4 different risk factors instead of 3, the discriminative power of the RFM was lower (AUROC 0.903) (Data not shown).

### Survival analyses

Survival analyses were conducted to further evaluate the discriminative power of AI and AI-RFM from prognostic point of view. First, survival between slow and rapid metastasis cohorts, as well as non-metastatic cohort was analyzed. Survival was higher in slow metastasis cohort up to approximately 4 years until slow metastasis cohort reached the level of rapid metastasis cohort with 50% of patients alive at 1.2 years for rapid metastasis and 3.4 years for slow metastasis cohort, when OS was considered and 1.3 and 4.1 years, respectively, when disease-specific survival (DSS) was considered (Supplementary Fig. 2). This notion emphasizes the importance of identifying especially mcSCCs, which metastasize rapidly.

**Figure 2:**
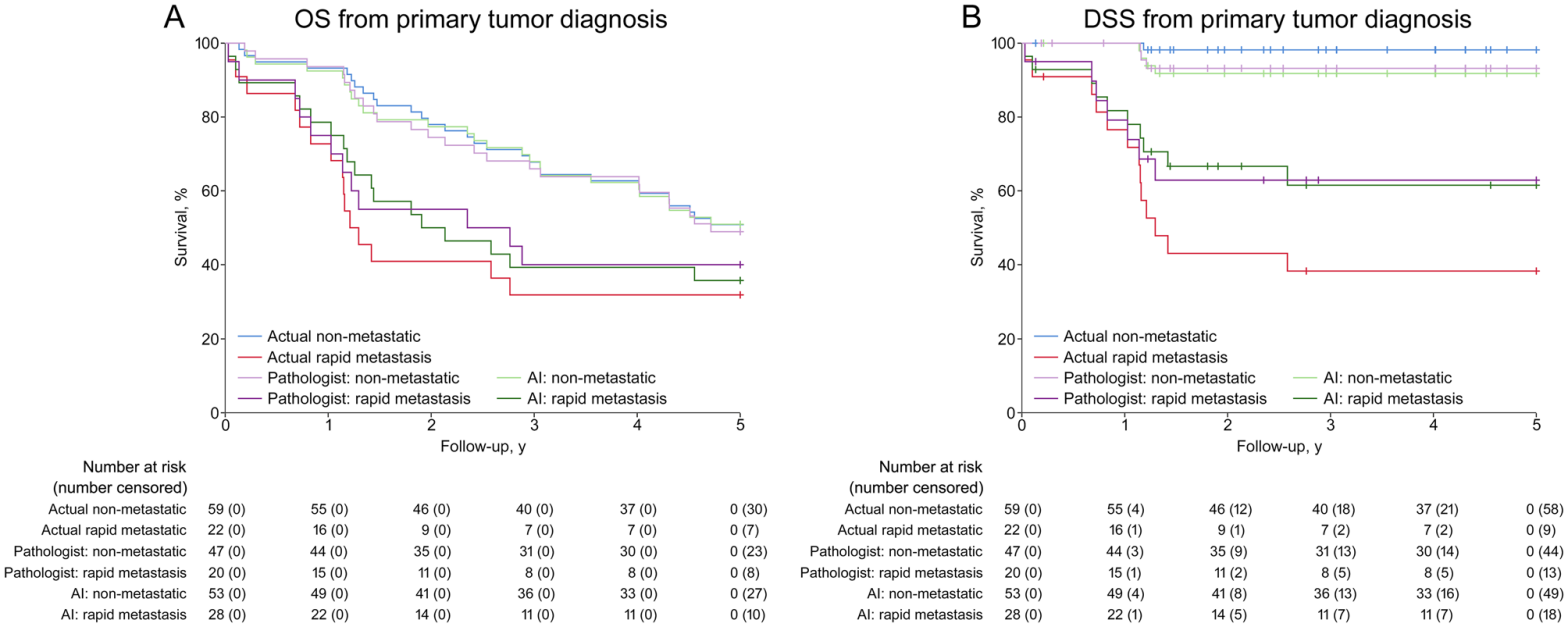
Kaplan–Meier overall survival (OS) and disease-specific survival (DSS) estimates calculated from the time of initial diagnosis of primary cSCC. (**A**) OS and (**B**) DSS estimates of actual non-metastasis (n = 59) and rapid metastasis (n = 22) cohorts in comparison with cohorts predicted by artificial intelligence (AI) as non-metastatic and rapid metastasis as well as cohorts predicted by pathologist as non-metastatic and rapid metastasis.

Next, the prognostic power of discrimination by both AI and dermatopathologist was analyzed. AI and pathologist provided nearly similar OS and DSS prediction (Fig. 2A,B).

**Figure 3:**
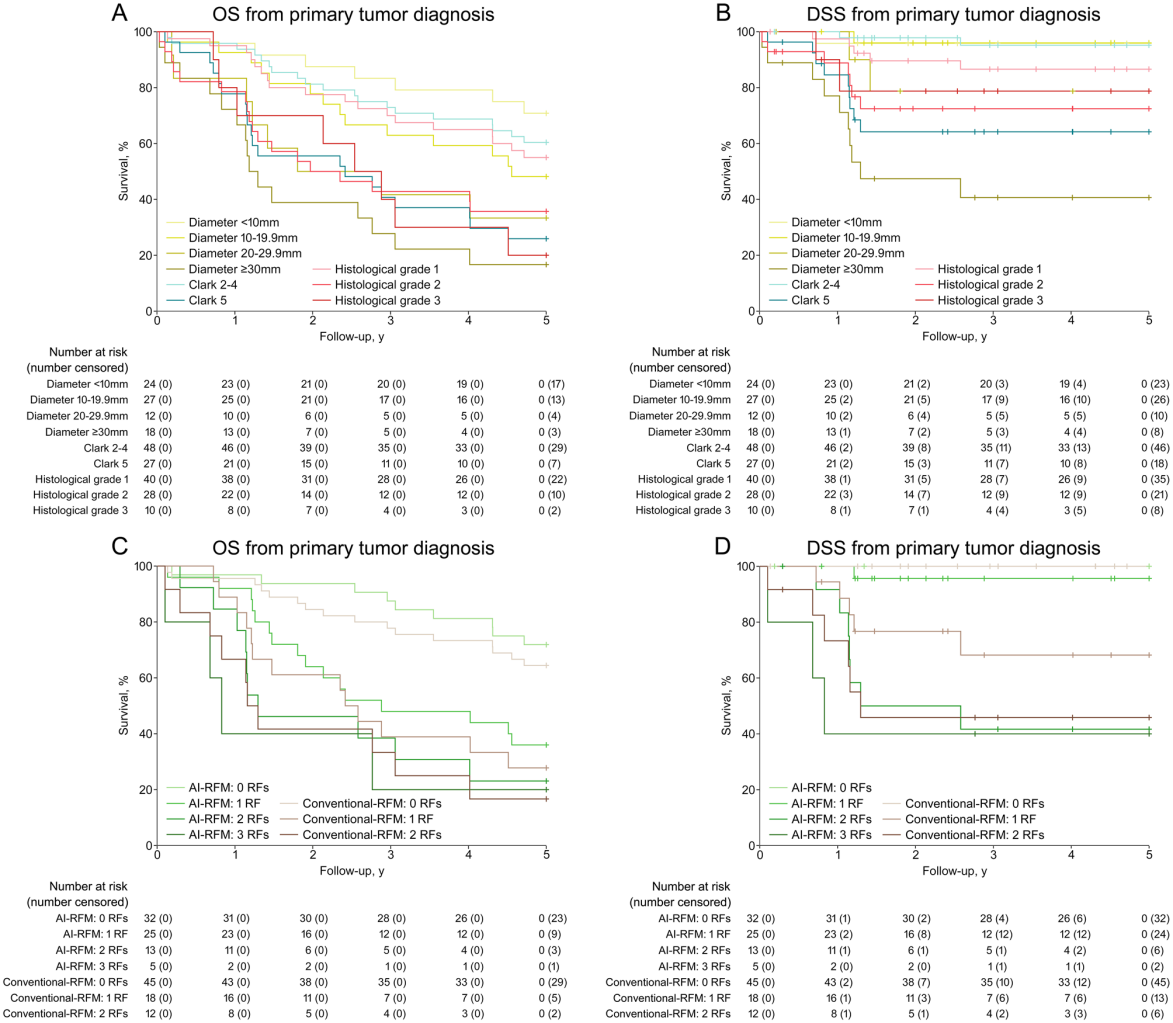
Kaplan–Meier overall survival (OS) and disease-specific survival (DSS) estimates calculated from the initial diagnosis of primary cSCC (rapid metastasis and non-metastatic cohorts, total tumor n = 81). (**A**) OS and (**B**) DSS estimates of cSCCs based on classification by primary tumor diameter, Clark’s level and histologic grade. OS (**C**) and DSS (**D**) estimates of cSCCs based on classification by artificial intelligence -risk factor model (AI-RFM) taking into account prediction by AI as metastatic, tumor diameter ≥ 30 mm and Clark’s level 5 as risk factors, and by conventional-RFM taking into account tumor diameter ≥ 30 mm and Clark’s level 5 as risk factors.

**Figure 4:**
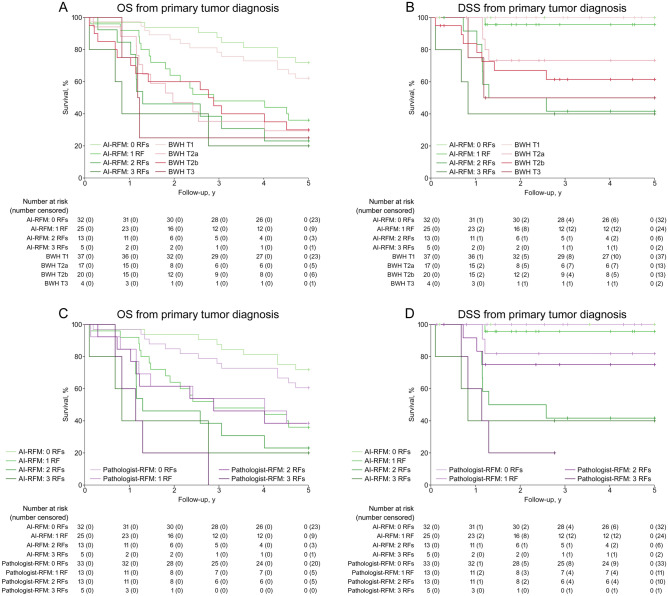
Kaplan–Meier overall survival (OS) and disease-specific survival (DSS) estimates calculated from the initial diagnosis of primary cSCC (rapid metastasis and non-metastatic cohorts, total tumor n = 81). (**A**) OS and (**B**) DSS estimates of cSCCs based on grouping by artificial intelligence- risk factor model (AI-RFM) taking into account prediction by AI as metastatic, tumor diameter ≥ 30 mm and Clark’s level 5 as risk factors and by Brigham and Women’s hospital (BWH) tumor staging. (**C**) OS and (**D**) DSS estimates of cSCCs based on classification by AI-RFM taking into account prediction by AI as metastatic, tumor diameter ≥ 30 mm and Clark’s level 5 as risk factors and by similar model utilizing prediction by pathologist instead of AI (pathologist-RFM).

In order to elucidate the feasibility of AI-RFM, the prognostic power of conventional histopathologic parameters was evaluated and visualized. Notably, the survival prediction by histological grade was inferior to diameter and Clark’s level, especially with respect to DSS (Fig. 3A,B). These observations support the inclusion of Clark’s level 5 and diameter ≥ 30 mm in AI-RFM as risk factors for prediction of metastasis in combination with AI (Fig. 3C,D). The AI-RFM provided survival prediction with excellent prognosis for patients with cSCC with 0 (5-year DSS estimate of 100%) or 1 (5-year DSS estimate of 95.7%) risk factors and poor prognosis for patients with cSCC with 2 (5-year DSS estimate of 41.7%) or 3 (5-year DSS estimate of 40.0%) risk factors (Figs. 3D, 4B,D). The discriminative power of AI-RFM was superior to conventional-RFM which included diameter ≥ 30 mm and Clark’s level 5 as risk factors with respect to DSS (Fig. 3D). Furthermore, the AI-RFM was compared to BWH tumor staging (Fig. 4A,B) and to the comparative RFM utilizing prediction by pathologist instead of AI (pathologist-RFM) (Fig. 4C,D). When DSS was considered the discriminative power of AI-RFM was superior to both BWH tumor staging (Fig. 4B) and pathologist-RFM (Fig. 4D). Notably, although the survival predictions by both AI and pathologist alone were almost identical (Fig. 2A,B), the AI-RFM was superior to pathologist-RFM (Fig. 4D) emphasizing the notion that the evaluation by AI is based on yet unestablished morphological features unlike the evaluation by pathologist. The superiority is based on the discrimination of cases by AI-RFM into good (0–1 risk factors) and poor prognosis (2–3 risk factors) and the lack of “grey zone” which turns out to be a problem with comparative RFMs and BWH staging.

## Discussion

To examine the ability of AI to recognize primary cSCCs that will develop metastasis, several approaches were tested with cross-validation. This is a clinically challenging task as to date there are no established biomarkers to predict the risk of metastasis or prognosis of primary cSCC. Conventional clinicopathological features are utilized in tumor staging systems, but these are unsatisfactory in predicting the risk of metastasis^7^.

Our results show that an experienced dermatopathologist and AI with limited training and testing data perform quite similarly in order to distinguish rapidly metastatic primary cSCCs from non-metastatic cSCCs. Although pathologists in clinical practice do not directly evaluate the risk of metastasis of primary cSCC, here the dermatopathologist was requested to do so. Notably, AI and pathologist had only one WSI available for analysis and no access to whole tissue material of the tumor or to clinical information from which the actual diameter or invasion depth of the whole tumor could have been deduced. By Pearson correlation (Supplementary Table 1) it appears that prediction by pathologist is based more on conventional histopathological features, such as invasion depth than the prediction by AI. This notion is further supported by similar survival prediction performance by AI and pathologist alone (Fig. 2A,B), in comparison to superior discriminative performance of AI-RFM to pathologist-RFM (Fig. 4D). These findings provide evidence, that the prediction by AI is based on yet unestablished morphological features or feature combinations. Additionally, these observations support our hypothesis, that these morphologic features appear in primary tumors temporally close to the time of metastasis.

The results obtained with the AI-RFM generated in this study show, that inclusion of AI algorithm in RFM improved the assessment of cSCC metastasis risk, as shown by AUROC of 0.917, which was superior to other RFMs and staging systems tested. Furthermore, the AI-RFM clearly differentiated cases with 0 or 1 risk factors from cases with 2 or 3 risk factors with respect to DSS prediction. This observation indicates that primary cSCCs with ≤ 1 risk factor should be considered as cases with low risk for metastasis and cSCCs with ≥ 2 risk factors as cases with high risk for metastasis when utilizing AI-RFM generated in this study.

To date, there are few published studies with similar study design in cancer field and no studies on metastasis risk of cutaneous cancers. A leave-one-out cross-validation accuracy of 80.77% was lately reported when predicting the risk of metastasis in pancreatic neuroendocrine tumors^21^. Furthermore, AUROC of 0.68 was achieved in prediction of lymph node metastasis of prostate cancer from primary tumor tissue utilizing CNN^22^. A third study harnessed CNN to predict lung cancer recurrence and metastasis from histopathological images and reached an AUROC of 0.79^23^. Our results provide evidence, that the AI prediction exploiting multifactorial RFM appears to be a more encouraging approach to the prediction of metastasis risk by AI.

It is conceivable, that further studies on AI and metastasis risk of primary cSCCs with larger cohorts for training and testing are warranted. It would be advisable to use tissue specimens scanned on same occasion or sufficiently large, preferably equally sized cohorts of samples scanned on different dates. Same approach is also recommended with usage of different scanners. Based on our findings, small subcohorts scanned with different scanners or on another date can generate bias and therefore identical scanning settings should be used. Furthermore, with larger cohorts it would be useful to further analyze the ability of AI algorithm to recognize both rapid and slow metastasis cases as well as biopsies and resections. Unfortunately, in this study with limited tissue specimens further analysis of the impact of above mentioned qualities as well as analysis of tissue samples at different stages of tumor progression could not be executed.

In summary, our results provide proof of concept, that there are certain yet unknown morphological features or feature combinations, which associate with the risk of cSCC metastasis and can be recognized by AI. Further studies are warranted in order to unveil these and to further develop AI algorithm as prognostic tool in combination with potential biomarkers and clinicopathological variables for the challenging assessment of the metastasis risk of cSCC.

## Materials and methods

### Ethical issues

The study was approved by The Ethics Committee of the Hospital District of Southwest Finland (187/2006) and Auria Biobank’s Scientific Steering Committee (AB15-9721). The research was carried out according to the Declaration of Helsinki. Registry study approval for collection and use of clinicopathological data was obtained from the Turku University Hospital Clinical Research Centre (18.11.2018; TO5/042/18). Informed consent was obtained from all subjects involved in the study.

### Research material

Hematoxylin and eosin-stained archived formalin-fixed and paraffin-embedded tissue specimens representing primary non-mcSCC and mcSCC tumors were scanned and digitalized into WSIs. Each tumor was obtained for diagnostic purpose from patients from the area served by Turku University Hospital. Eventually, one tumor per patient and one WSI representing each cSCC was utilized to train and test the AI algorithm, and in blind analysis by dermatopathologist. Altogether 45 mcSCC from 45 and 59 non-mcSCCs from 59 individual patients, who did not develop cSCC metastasis during at least 5-year follow-up were included in the analyses. mcSCCs were further divided into two subcohorts: one with primary mcSCCs, which developed metastasis within 180 days (rapid metastasis cohort) (n = 22) and another with primary mcSCCs that developed metastasis after 180 days from the initial diagnosis of primary tumor (slow metastasis cohort) (n = 23). Clinical, histopathological, follow-up and demographic data were gathered manually from patient records and pathology reports (Tables 1,2). All mcSCCs developed at least one nodal metastasis and part also distant metastases.

In survival analyses death was the primary clinical endpoint. The exact cause of death was rarely reported and autopsies were infrequently performed, but the cause of death could be deduced from patient records with acceptable reliability by clinician (JSK). Both unambiguous OS and deduced DSS were utilized in survival analyses. Survival time was calculated from the date of initial diagnosis of the primary cSCC with 5 years of follow-up. Death represented the end of follow-up.

Tissue specimens (slides) were scanned with 3DHistech scanner (3DHistech, Budapest, Hungary) and annotated using CaseViewer (3DHistech, Budapest, Hungary). The WSIs representing cSCCs were manually annotated. Annotated area included whole tumor represented on the WSI, including tumor cells, intratumoral and peritumoral stroma, as well as intratumoral inflammatory cells (Supplementary Fig. 3). Additionally, manual exclusions were made if artefacts were detected in annotated tumor area. These annotated areas of the WSIs were used to train and validate the neural networks.

As WSIs are too large to feed any ML model, the images were cut into 1024 × 1024 and 512 × 512 pixel tiles at the largest zoom level (20 ×) from the annotated tumor areas. The tissue content of the tiles was further analyzed by using a combination of binary and Otsu thresholding: a tile was considered valid if it contained more than 50% of tissue pixels as defined by the thresholding algorithm. Similarly, a tile was considered a tumor tile if it contained more than 50% pixels from the tumor annotation mask. All tiles inherited the cohort label from the original slide. Every tile was then resized into 299 × 299 pixels before training the algorithms.

### Training setup

We analyzed the task of distinguishing mcSCCs from non-mcSCCs as a binary classification problem on the level of a single tumor tile. Each tumor tile was assigned to a single cohort and the binary classification problem was to classify the tiles into these two classes. As the data set is relatively small we approached the task with cross-validation, in which data is sectioned into subsets and one subset at a time is used for testing and rest for training. Thus, several models are trained and the performance of each model is tested with alternating validation subset.

Final reported results of the rapid metastasis -AI-model are based on the ResNet18 architecture with a custom head consisting of average pooling and two dense layers with heavy dropout to combat overfitting. More complex ResNet models (ResNet50) were also tested but were more prone to overfitting, which lead to the choice of a simpler model for the final rapid metastasis -AI-model. The models were trained using threefold cross-validation (3-CV) with different extraction tile sizes until the final rapid metastasis -AI-model in which fourfold cross-validation (4-CV) was used. The loss function used was the binary cross-entropy loss. In training, it was confirmed that the tiles from a given patient and slide were exclusively sampled into one of the cross-validation-folds to prevent the leakage of information between the training and validation sets. With every fold the model was trained 10 or 20 epochs.

### Visualization of the results

**Figure 5:**
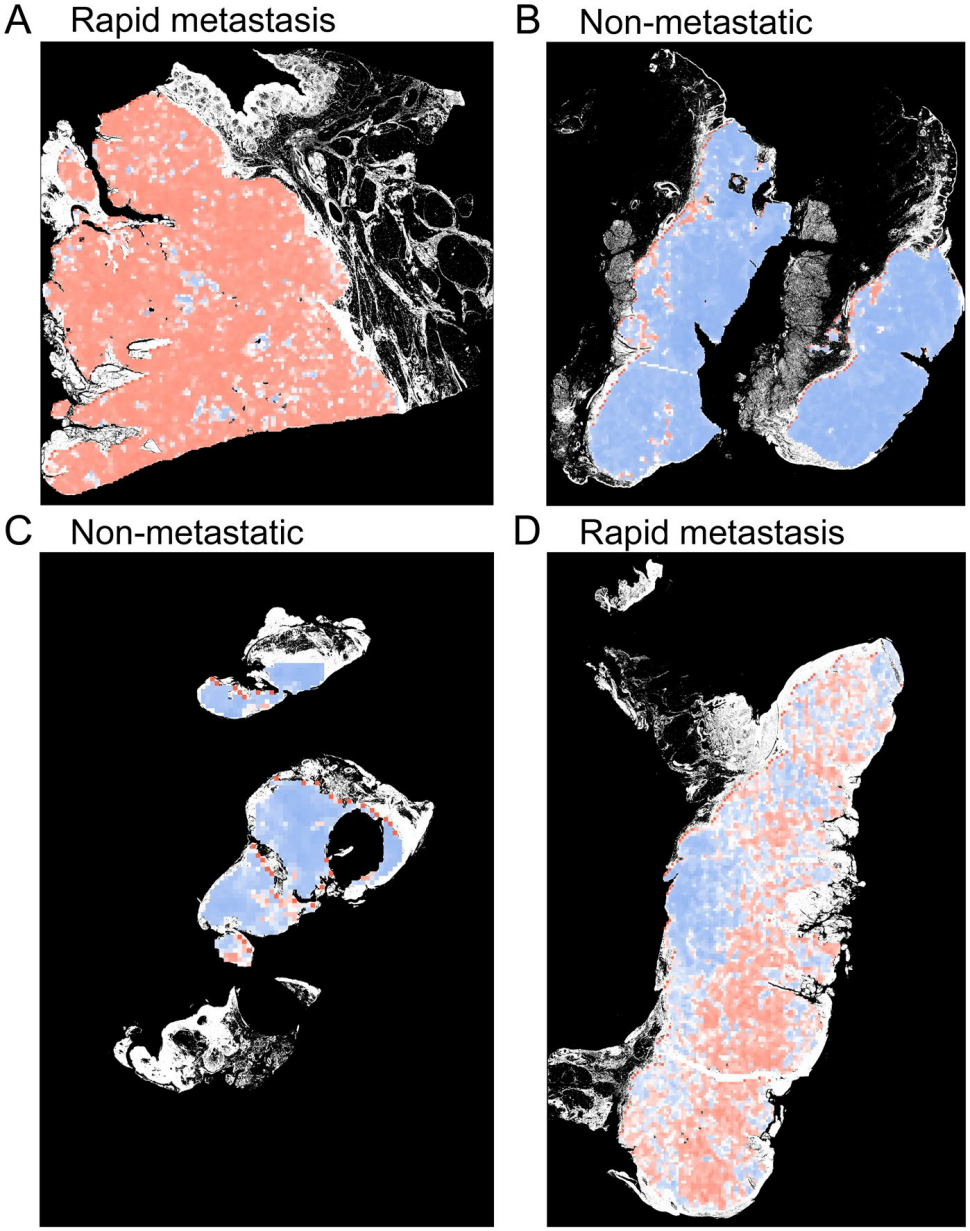
Probability maps of annotated whole slide images analyzed by artificial intelligence (AI). The color in the probability map indicates the predicted metastasis score by AI on tile level in annotated tumor area. Red color represents high and blue color low score i.e. red color indicates tiles analyzed as metastatic and blue color tiles analyzed as non-metastatic by AI algorithm. White color on the edge of the slides represents excluded tissue outside manual annotations. (**A**) and (**D**) represent rapid metastasis and (**B**) and (**C**) non-metastatic cSCCs that were classified correctly on slide level by AI.

In addition to analyzing the cross-validation accuracy and loss, we created the out-of-the-fold (OOF) tile level receiver operating characteristic (ROC) curves and calculated area under the curve (AUC) values. The OOF tile level predictions were also mapped to slide level. Namely, each tile and the corresponding location in the WSI was assigned a prediction probability between 0 and 1 for developing a metastasis. This probability was scaled to [0,100] and then shifted to the interval [− 50, 50] for each tile. The scores on WSI were then spatially smoothed using a median filter with window size 2. This meant that a window of 2 × 2 tiles was moved along the slide and the tile value was replaced with the median of the tile values within the window. The idea was to remove noise according to our hypothesis, that the features representing metastasis risk would vary smoothly across the tissue slide. To date, there is no way to scientifically select a correct size for the smoothing window. Our selection was based on visualizations of the results. Probability maps of the annotated tumors were generated for metastasis score visualization (Fig. 5).

**Figure 6:**
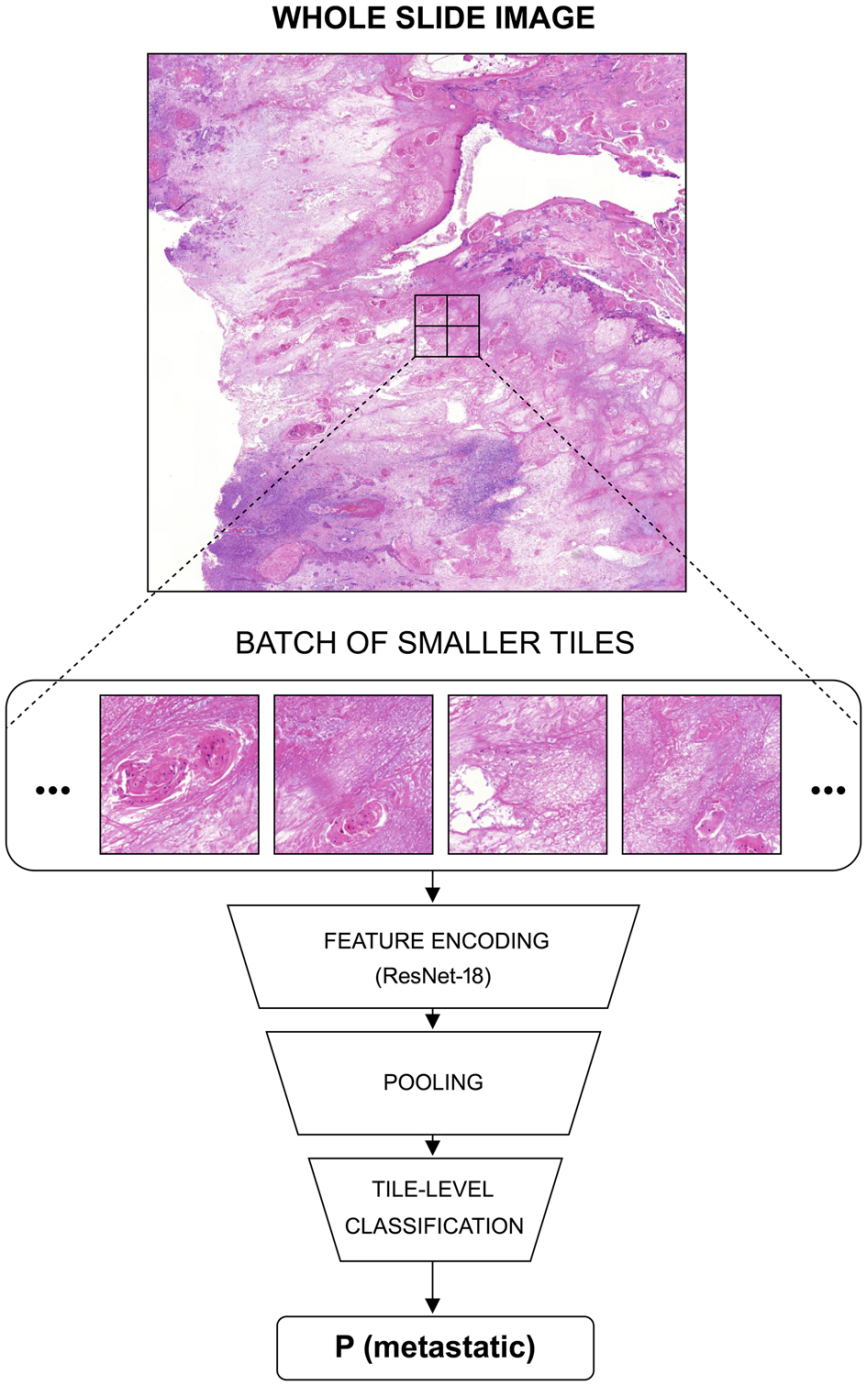
The rapid metastasis -AI-model workflow. The whole slide images are divided into small tiles. The tiles are assigned the binary yes/no tumor labels based on the annotations. The tumorous tiles are further labeled based on the metadata to yes/no rapid metastasis. This is done for all of the WSI images. The ResNet-18 model is trained to classify the tiles according to the labels. Batches of tiles are fed to the model, which then learns to extract relevant visual features (feature encoding) from them and produce a classification. Finally, the confidence scores "P(metastatic)" are aggregated to produce whole slide level results.

After the previous steps, where the OOF tile level predictions were accumulated to slide level, a simple majority vote of the scaled predictions was performed to determine the predicted label of the WSI and the tumor by assessing the mean slide level score. We took 0 as the decision threshold to discriminate between the low/high metastasis risk tumors and visualized the slide level results in ROC curves with AUC scores and summary confusion matrices. The workflow from WSI input to tile level result is visualized in Fig. 6.

### AI-models and hypotheses

In our initial approach we used single tile -AI-model, in which individual tiles served as input. Hypothetically, as most of the cells are the offspring of the cancerous cells and inherit the genetic alterations of the first generation of tumor cells, most tiles should represent the possible differences between the metastatic and non-metastatic tumors. It was noted that this classification algorithm is easy to implement and it is easy to aggregate and visualize the single tile predictions at slide level, but the model is prone to label noise. Another approach was to focus on the invasive front of the tumor (invasive front -AI-model). Hypothetically, metastatic characteristics of the tumor are more likely visualized in the invasive front and focusing on this area was expected to lead to less noisy training labels. Third approach was to use stack of tiles (multi-tile -AI-model) as an input data in order to reduce the noise in the labels. Instead of just one tile, randomly selected sample of stack of n tiles from the same WSI was used as the input of the classifier. In above mentioned approaches ResNet50 architecture was used with 3-CV and single tiles from annotated WSIs representing the two cohorts (primary mcSCCs and primary non-mcSCCs) were used as input data. In multi-tile -AI-model instead of single tile a stack of tiles and in invasive front -AI-model only tiles adjacent to the edge of the annotation (either inside and/or outside of the annotated area) from annotated WSIs were used as input data.

Due to previous notion of rapid development of metastasis^3^ we subdivided the mcSCC cohort into cases that metastasize rapidly and slowly. Furthermore, we hypothesized that features characteristic for the metastatic tumor would be more prominent or more probably already present at the date of tissue specimen in primary tumors, which metastasize rapidly. Therefore, only tumors, which metastasized rapidly were utilized in the rapid metastasis -AI-model. Both stack of tiles and single tiles from annotated WSIs representing two cohorts (primary non-mcSCCs and primary rapid mcSCCs) were used as input data. Fourfold cross-validation and ResNet18 architecture were ultimately used. To further prevent overfitting into the more exclusive dataset, we used a “zoomed-in” approach in which 512 × 512 pixels instead of 1024 × 1024 pixels tiles were used.

Regarding technical execution of the study, during repeated runs of 3 × or 4 × CV analysis on all of the available slides for training, it seemed that depending on the choice of the CV split, the results varied considerably. Often it seemed that one fold was much more difficult for the model to analyze than the others, leading to considerably lower AUROC values. We took this as a sign that the dataset contained some examples that were difficult for the model to analyze, for example by being very atypical cases of rapid metastasis tumors. More careful analysis of the metadata revealed, however, that the difficult cases appeared in folds where the slide data had been scanned in 2020 instead of 2016. Most of the rapid metastasis cases were scanned earlier because various research projects have been conducted with the samples in the past. The scanner used was the same, but we hypothesized that the scanning software, image packing algorithms or the physical components of the scanner itself could have been updated affecting the image colors, noise patterns or other qualities. We used heavy color augmentations in training of the algorithm, but to study the possible effect of slides scanned at different times we tried and restricted the data to old samples only. This reduced the size of the dataset available, but was useful in ruling out at least one more possible source of error. This affected the AUROC results somewhat and brought the average AUROC value up.

### Blinded assessment by pathologist

For comparison purposes, every WSI included in the final rapid metastasis -AI-model was analyzed by experienced dermatopathologist (LT). Only tissue sample ID and information, whether the specimen represented biopsy or resection was provided to the pathologist in accordance with access to CaseCenter folder including 81 WSIs representing non-mcSCCs and rapid metastasis mcSCCs without the knowledge of the proportion of cases. The histological criteria used by the pathologist for predicting metastasis in this series included invasion depth, histological grade, tumor size, perineural invasion and also the subjective interpretation of the pattern of invasion (large vs. small nests/single cells). Although for instance invasion depth is a part of staging systems, no staging system as such was used. The pathologist also had the option not to classify the specimen into the cohorts, if assessment was not possible, as was the case with biopsy samples.

### Statistical analysis

All statistical analyses were conducted using IBM SPSS Statistics for Windows, version 25.0 (IBM Corp., Armonk, NY, USA). Bidirectional *p *values < 0.05 and 95% confidence intervals (95% CIs) of odds ratios (ORs) not including 1.00 were considered statistically significant. Baseline tumor characteristics were analyzed using descriptive statistics mainly crosstabs and frequency tabulation. Statistical analyses were conducted with Pearson χ^2^ test and Fisher's exact test. Binary logistic regression analyses with 95% CIs were performed in order to determine ORs regarding the risk of metastasis. For every risk factor and risk factor combination an AUROC was calculated in order to further visualize results in relation to AI prediction. Pearson correlation was conducted in order to visualize multicollinearity and to examine whether predictions correlated with some of the clinicopathological variables. The Kaplan–Meier method was applied to generate survival estimate curves and define survival probabilities.

## Supplementary Information

Supplementary Information.

## Data Availability

The data collected during this study is patient data obtained under Ethical Committees approval and cannot be shared.
