# Supplementary material for: Identification of metastatic primary cutaneous squamous cell carcinoma utilizing artificial intelligence analysis of whole slide images
Source: Sci Rep. 2022 Jun 14;12:9876. doi: 10.1038/s41598-022-13696-y (PMC9197840; doi:10.1038/s41598-022-13696-y)
Supplement: Supplementary file 1 — Supplementary Information. [file 41598_2022_13696_MOESM1_ESM.pdf]

Identification of metastatic primary cutaneous squamous cell carcinoma utilizing artificial intelligence analysis of whole slide images

Jaakko S. Knuutila [1,2], Pilvi Riihilä[1,2], Antti Karlsson[3], Mikko Tukiainen[3], Lauri Talve[4], Liisa Nissinen[1,2] and Veli-Matti Kähäri[1,2]

[1]Department of Dermatology, University of Turku and Turku University Hospital, Turku, Finland. [2]FICAN West Cancer Research Laboratory, University of Turku and Turku University Hospital. [3]Auria Biobank, University of Turku and Turku University Hospital, Turku, Finland. [4]Department of Pathology, University of Turku and Turku University Hospital, Turku, Finland

Supplementary Material

Supplementary Figure 1 Confusion matrices representing slide level results of rapid metastasis - AI-model

Supplementary Figure 2 Kaplan–Meier overall survival (OS) and disease-specific survival (DSS) estimates calculated from the initial diagnosis of primary cSCC

Supplementary Figure 3 Examples of whole slide images (WSIs) with manual annotations

Supplementary Table 1 Pearson correlations

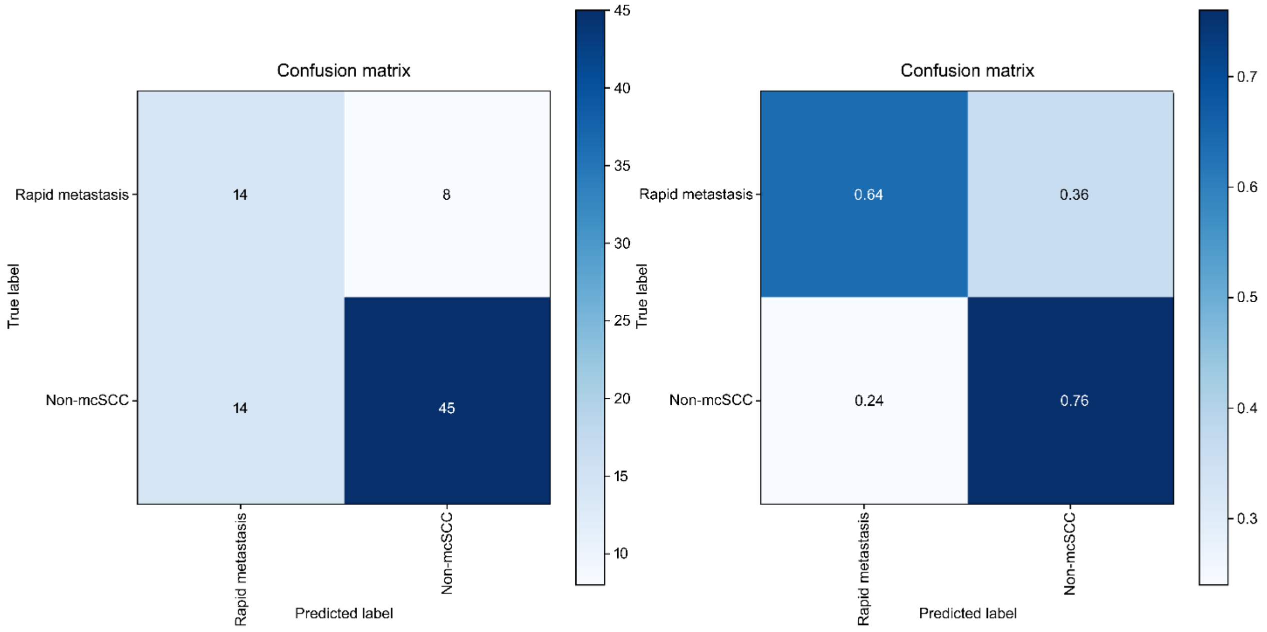

Supplementary Figure 1: Confusion matrices representing slide level results of rapid metastasis -AI-model. Confusion matrices show the pair-wise comparison. In the confusion matrices the diagonal shows the number (left) and percentage (right) of cases correctly classified.

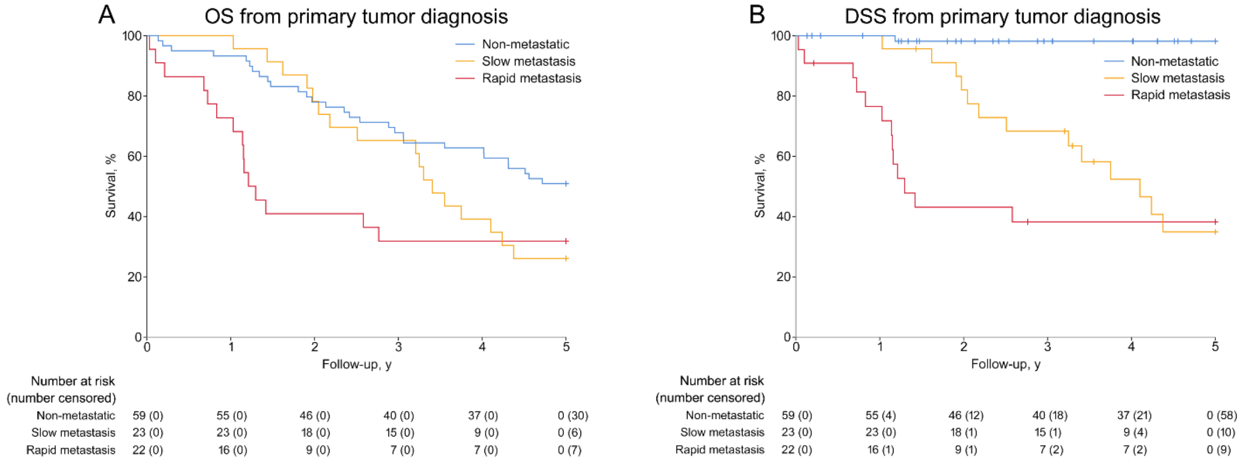

Supplementary Figure 2: Kaplan–Meier overall survival (OS) and disease-specific survival (DSS) estimates calculated from the initial diagnosis of primary cSCC. OS (**A**) and DSS (**B**) estimates of actual non-metastatic (n=59), slow metastasis (n=23) and rapid metastasis (n=22) cohorts.

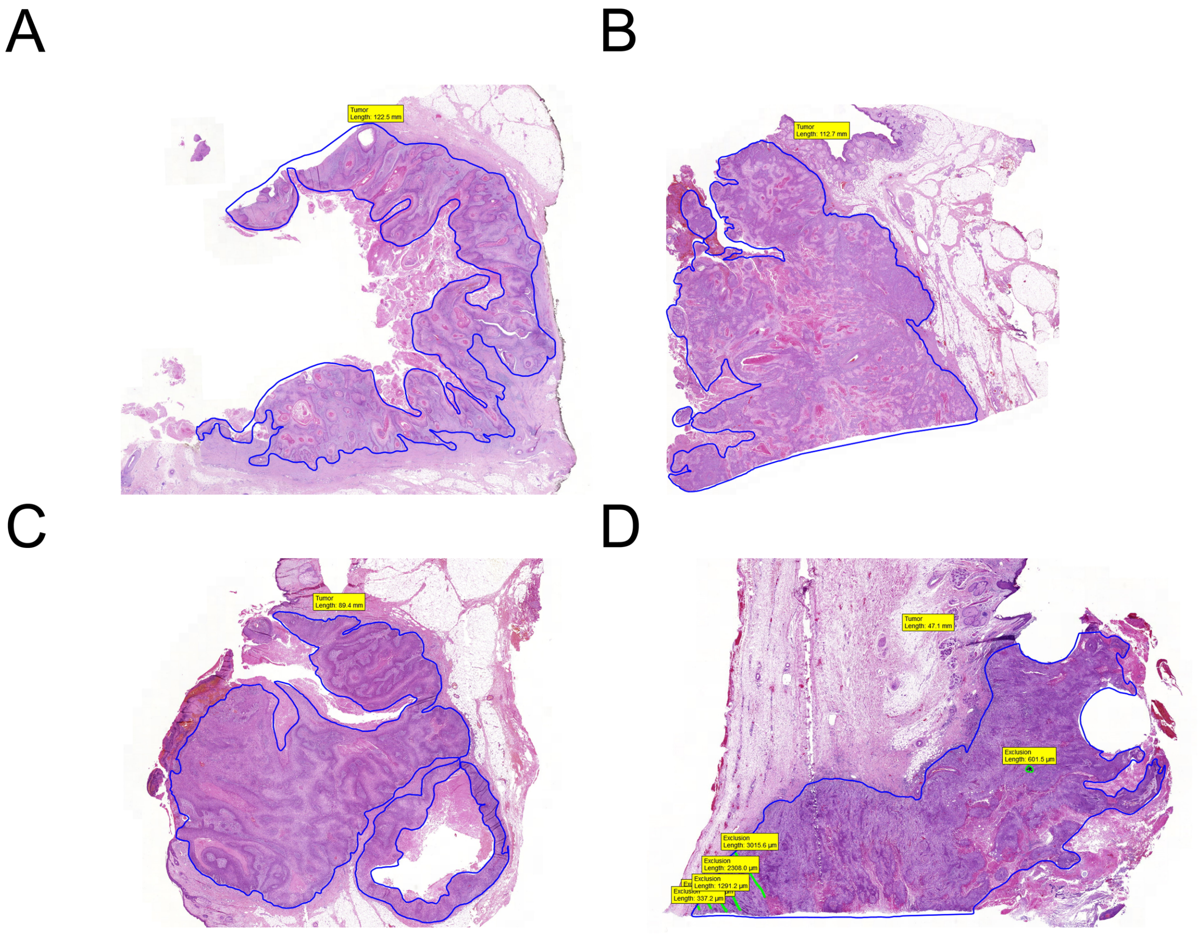

Supplementary Figure 3: Examples of whole slide images (WSIs) with manual annotations. Annotated tumor areas include tumor cells, intratumoral and peritumoral stroma as well as intratumoral inflammatory cells and excludes normal skin and other normal tissues surrounding the tumor as shown in the figure. (**A, B**) primary metastatic cSCCs that metastasize rapidly. (**C, D**) primary non-metastatic cSCCs. Blue represents annotation line and green line exclusions due to artefacts in whole slide image.

Correlations

		Invasion beyond fat	AJCC-8	BWH	Prediction by AI	Clark's level	Gender	Location	Prediction by pathologist	Diameter	Gradus	Age	Tissue specimen (biopsy/resection)	Perineural invasion	Lymphovascular invasion	Number of local recurrences
Beyond Fat	Pearson Correlation	1	.838**	.780**	0,189	.553**	-0,161	-0,017	.287*	.441**	.227*	0,088	-0,147	-0,071	0,165	.266*
	Sig. (2-tailed)		0,000	0,000	0,093	0,000	0,153	0,883	0,020	0,000	0,047	0,437	0,192	0,529	0,143	0,017
	N	80	80	78	80	75	80	80	66	80	77	80	80	80	80	80
AJCC-8	Pearson Correlation	.838**	1	.879**	.329**	.658**	-0,162	-0,046	.380**	.606**	.340**	0,119	-0,139	-0,091	.227*	.301**
	Sig. (2-tailed)	0,000		0,000	0,003	0,000	0,150	0,685	0,002	0,000	0,002	0,293	0,217	0,423	0,043	0,007
	N	80	80	78	80	75	80	80	66	80	77	80	80	80	80	80
BWH	Pearson Correlation	.780**	.879**	1	.256*	.645**	-0,061	-0,002	.494**	.664**	.540**	0,124	-0,206	-0,105	.232*	.310**
	Sig. (2-tailed)	0,000	0,000		0,024	0,000	0,599	0,988	0,000	0,000	0,000	0,280	0,071	0,360	0,041	0,006
	N	78	78	78	78	73	78	78	65	78	77	78	78	78	78	78
Prediction by AI	Pearson Correlation	0,189	.329**	.256*	1	0,021	-0,073	-0,005	0,215	.226*	0,092	-0,138	-.242*	-0,081	0,132	0,138
	Sig. (2-tailed)	0,093	0,003	0,024		0,855	0,515	0,967	0,080	0,043	0,426	0,218	0,029	0,471	0,239	0,221
	N	80	80	78	81	75	81	81	67	81	78	81	81	81	81	81
Clark's level	Pearson Correlation	.553**	.658**	.645**	0,021	1	-0,098	-0,004	.415**	.587**	.504**	0,090	0,119	-0,087	0,048	-0,027
	Sig. (2-tailed)	0,000	0,000	0,000	0,855		0,405	0,971	0,001	0,000	0,000	0,444	0,310	0,457	0,681	0,818
	N	75	75	73	75	75	75	75	64	75	73	75	75	75	75	75
Gender	Pearson Correlation	-0,161	-0,162	-0,061	-0,073	-0,098	1	.296**	0,122	0,023	0,088	0,153	-0,102	-0,079	-0,139	-0,116
	Sig. (2-tailed)	0,153	0,150	0,599	0,515	0,405		0,007	0,326	0,835	0,442	0,173	0,365	0,483	0,217	0,304
	N	80	80	78	81	75	81	81	67	81	78	81	81	81	81	81
Location	Pearson Correlation	-0,017	-0,046	-0,002	-0,005	-0,004	.296**	1	-0,089	0,026	-0,110	0,003	-0,026	-0,010	-0,108	-0,139
	Sig. (2-tailed)	0,883	0,685	0,988	0,967	0,971	0,007		0,476	0,815	0,338	0,980	0,817	0,932	0,338	0,217
	N	80	80	78	81	75	81	81	67	81	78	81	81	81	81	81
Prediction by pathologist	Pearson Correlation	.287*	.380**	.494**	0,215	.415**	0,122	-0,089	1	0,228	.415**	0,032	0,164	-0,080	.269*	0,233
	Sig. (2-tailed)	0,020	0,002	0,000	0,080	0,001	0,326	0,476		0,064	0,001	0,797	0,184	0,518	0,028	0,058
	N	66	66	65	67	64	67	67	67	67	65	67	67	67	67	67
Diameter	Pearson Correlation	.441**	.606**	.664**	.226*	.587**	0,023	0,026	0,228	1	.352**	0,093	-0,089	-0,030	-0,111	-0,133
	Sig. (2-tailed)	0,000	0,000	0,000	0,043	0,000	0,835	0,815	0,064		0,002	0,409	0,432	0,793	0,325	0,236
	N	80	80	78	81	75	81	81	67	81	78	81	81	81	81	81
Gradus	Pearson Correlation	.227*	.340**	.540**	0,092	.504**	0,088	-0,110	.415**	.352**	1	0,085	0,031	-0,100	0,110	0,146
	Sig. (2-tailed)	0,047	0,002	0,000	0,426	0,000	0,442	0,338	0,001	0,002		0,460	0,789	0,384	0,340	0,203
	N	77	77	77	78	73	78	78	65	78	78	78	78	78	78	78
Age	Pearson Correlation	0,088	0,119	0,124	-0,138	0,090	0,153	0,003	0,032	0,093	0,085	1	0,090	-0,192	0,001	0,167
	Sig. (2-tailed)	0,437	0,293	0,280	0,218	0,444	0,173	0,980	0,797	0,409	0,460		0,427	0,086	0,991	0,137
	N	80	80	78	81	75	81	81	67	81	78	81	81	81	81	81
Tissue specimen (biopsy/resection)	Pearson Correlation	-0,147	-0,139	-0,206	-.242*	0,119	-0,102	-0,026	0,164	-0,089	0,031	0,090	1	0,044	-0,113	0,029
	Sig. (2-tailed)	0,192	0,217	0,071	0,029	0,310	0,365	0,817	0,184	0,432	0,789	0,427		0,694	0,315	0,794
	N	80	80	78	81	75	81	81	67	81	78	81	81	81	81	81
Perineural invasion	Pearson Correlation	-0,071	-0,091	-0,105	-0,081	-0,087	-0,079	-0,010	-0,080	-0,030	-0,100	-0,192	0,044	1	-0,022	-0,025
	Sig. (2-tailed)	0,529	0,423	0,360	0,471	0,457	0,483	0,932	0,518	0,793	0,384	0,086	0,694		0,846	0,824
	N	80	80	78	81	75	81	81	67	81	78	81	81	81	81	81
Lymphovascular invasion	Pearson Correlation	0,165	.227*	.232*	0,132	0,048	-0,139	-0,108	.269*	-0,111	0,110	0,001	-0,113	-0,022	1	0,172
	Sig. (2-tailed)	0,143	0,043	0,041	0,239	0,681	0,217	0,338	0,028	0,325	0,340	0,991	0,315	0,846		0,124
	N	80	80	78	81	75	81	81	67	81	78	81	81	81	81	81
Number of local recurrences	Pearson Correlation	.266*	.301**	.310**	0,138	-0,027	-0,116	-0,139	0,233	-0,133	0,146	0,167	0,029	-0,025	0,172	1
	Sig. (2-tailed)	0,017	0,007	0,006	0,221	0,818	0,304	0,217	0,058	0,236	0,203	0,137	0,794	0,824	0,124	
	N	80	80	78	81	75	81	81	67	81	78	81	81	81	81	81

**. Correlation is significant at the 0.01 level (2-tailed).

*. Correlation is significant at the 0.05 level (2-tailed).

Supplementary Table 1: Pearson correlations.

AI: artificial intelligence; AJCC-8: The eight edition of American joint committee on cancer tumor staging; BWH: Brigham and Women's Hospital tumor staging; Gradus: histological grade; Location: primary tumor location
